# 360 degree perspective on allergic rhinitis management in Italy: a survey of GPs, pharmacists and patients

**DOI:** 10.1186/s12948-015-0029-5

**Published:** 2015-11-02

**Authors:** G. Walter Canonica, Massimo Triggiani, GianEnrico Senna

**Affiliations:** Respiratory and Allergy Clinic, DIMI-Department of Internal Medicine, University of Genoa, IRCCS Aou San Martino, Largo Rosanna Benzi 10, 16132 Genoa, Italy; Division of Allergy and Clinical Immunology, University of Salerno, Salerno, Italy; Allergy Unit, University Hospital Verona, Verona, Italy

**Keywords:** Allergic rhinitis, Burden, Italy, Physician, Pharmacist, Patient, Treatment, Survey

## Abstract

**Background:**

General practitioners (GPs), community pharmacists and allergic rhinitis (AR) patients in Italy were surveyed in order to gain insight from all three perspectives into the diagnosis, management and burden of AR in Italy.

**Methods:**

General practitioners and pharmacists (n = 100 for each) were surveyed by telephone; questions related to overall practice and to last AR patient seen. Patients (n = 552) completed a questionnaire after visiting specialist allergy centres. Questions related to diagnosis and treatment, degree of everyday limitation from AR, and satisfaction with treatment. The data were analysed descriptively.

**Results:**

Allergic rhinitis was managed mainly by GPs, who reported making the diagnosis themselves in 68 % of cases; rhinorrhea (64 %), sneezing (57 %) and congestion (49 %) were the symptoms most frequently taken into account. Limitation from AR on everyday life was rated 6.2 out of 10 by GPs. Pharmacists most often considered eye tearing (54 %) in their diagnosis. Almost half of GPs (49 %) and 87 % of pharmacists were unaware of the Allergic Rhinitis and its Impact on Asthma (ARIA) guidelines. The most commonly reported prescribed treatments by GPs were branded mometasone furoate, desloratadine, ebastine and generic mometasone; 21 % prescribed homeopathic products occasionally. On average, GPs remembered that their last patient case had moderate/severe disease, was prescribed anti-histamine monotherapy (37 % of cases), and did not change prescription (78 %). Pharmacists recommended an antihistamine for 56 % of clients who asked for advice, and a nasal decongestant for 21 %. Patients rated limitation from AR on everyday life as 5.7/10. 55 % reported using multiple therapies, and 43 % were not satisfied or weakly satisfied with their current treatment. Patients’ main expectation for the future was to succeed in managing their AR symptoms (45 %), while 22 % hoped for a definitive cure. Many patients (61 %) were concerned their health would deteriorate.

**Conclusions:**

Allergic rhinitis is largely managed by GPs in Italy, with pharmacists also playing a role, yet awareness of the ARIA guidelines among these groups is low. Patient satisfaction with treatment is moderate or low. New more effective treatments are needed to improve AR management in Italy. Allergy education programs need to be better targeted to GPs and pharmacists, and communication with patients regarding symptom control must be improved.

**Electronic supplementary material:**

The online version of this article (doi:10.1186/s12948-015-0029-5) contains supplementary material, which is available to authorized users.

## Background

Allergic rhinitis (AR) is the ‘poor relation’ of the allergy world, firmly placed behind asthma and atopic dermatitis in order of importance. The burden of AR is often ignored, but it is associated with a significant negative impact on productivity (comparable with that of heart disease and diabetes) [1] and high costs; estimated in Italy at €210.43/patient/year, a total cost to the Italian economy of approximately €2.4 billion/year [2–4]. AR has even been associated with an increased risk of having a road traffic accident (similar to that associated with having a blood alcohol concentration of 0.05 %, the legal limit in many countries) [5]. The Allergic Rhinitis and its Impact on Asthma (ARIA) guidelines are the recognised worldwide authority on AR diagnosis and management [6, 7]. The ultimate aim of the guidelines is to achieve AR control. However, disease control in AR remains an elusive concept, and indeed has not been fully defined.

There are several reasons why AR remains poorly controlled for many patients, including the complexity of its underlying pathology, lack of a universally accepted ‘control-concept’ and control language [8], a shifting landscape of severity [9, 10], sensitisation patterns [11, 12] and phenotypes [13, 14], and insufficiently effective and fast-acting treatments [15, 16]. Currently considered first-line therapies (even multiple therapies) do not consistently offer optimal symptomatic relief for many AR patients [17, 18]. Factors relating to patients and healthcare providers (HCPs) also contribute, and it is these factors that we examine in the current survey, specifically from the Italian viewpoint.

In Italy the prevalence of AR is high, estimated at 18.9 % of the population and rising [2, 3]. As in most jurisdictions, treatment patterns may be influenced by reimbursement regulations. Antihistamines are reimbursed in all regions of Italy for chronic (>3 months) treatment of AR but not for on-demand therapy. Nasal corticosteroids are not reimbursed, except in Tuscany.

The burden of AR in Italy has been assessed in the past, but these surveys have focused on a particular patient population (e.g. children) [19] or else the data from Italy has been combined with that of other European countries [9] and the United States [20]. However, in order to assess the true burden of AR in Italy and describe the current AR landscape, a ‘360-degree’ perspective should be employed. Such a perspective includes the viewpoint of the general practitioner (GP) who diagnoses AR and prescribes medication, that of the pharmacist who dispenses medication and advises on AR treatments and referrals, and that of the patient who lives with the symptomatic burden and the consequences of medication and referral choices.

The aim of the current survey was to fill gaps in our knowledge on the burden and management of AR in Italy. We sought to collect a global perspective on AR management in Italy for the first time, by surveying GPs, pharmacists and patients from diagnosis to treatment and beyond. Specifically, the survey aimed to compose a picture of how AR is diagnosed and treated by GPs and pharmacists and to assess concerns and expectations for the future from the patient perspective.

## Methods

Separate surveys were conducted of GPs, community pharmacists and AR patients. Survey questions are listed in Additional file 1. The GP and pharmacist surveys were carried out using computer-assisted telephone interviews, with a sample of 100 individuals from each category. The GPs and pharmacists participating in the study were randomly selected from a national database and were considered representative of their professional group working within the different geographical regions of Italy. Any GPs or pharmacists who had participated in market research within the previous 6 months were not enrolled. Interviews were carried out between 4th September 2013 and 24th September 2013.

### GP survey

Information was collected on participant demographics, length of practice, number of patients with AR seen each month, practice in relation to diagnosis and referral of AR, symptoms considered when diagnosing AR, pathologies and comorbidities associated with AR, practice relating to the treatment of AR patients (including most frequently prescribed treatments and modalities), and awareness of the ARIA guidelines. GPs were also asked how much AR limited their patients’ everyday life (on a scale from 1 to 10 where 1 = not at all and 10 = very limiting).

### Pharmacist survey

The information collected from pharmacists was similar to that described above for GPs, except that beliefs about underlying inflammation and relationship to asthma were surveyed instead of more open questions on pathology and comorbidity, and questions on treatment patterns were broken down into doctors’ prescriptions, cases where the patient asked for a recommendation, and cases where the patient chose their own treatment. Pharmacists were not questioned on the degree of limitation suffered by patients.

The GP and pharmacist surveys referred to participants’ practice in general, except for a section that questioned them specifically on the last AR patient seen. This ‘last patient case’ approach enabled more specific questions to be asked about the case severity and treatment approach, and was also expected to provide a greater degree and accuracy of memory recall.

### Patient survey

Patients were recruited from multiple specialist allergy centres throughout Italy. Eligible patients were those with AR attending participating centres for the first time. Patients were surveyed via a self-completed written questionnaire, which was completed by a total of 552 individuals. Questions covered duration of symptoms, time since diagnosis, role of different categories of HCP in patient’s diagnosis and treatment, sources of information about AR, treatments used, satisfaction with treatment, degree of limitation from AR in everyday life (1–10 scale as for GP survey), beliefs on links with other pathologies, chief concerns relating to AR, and expectations of future treatment.

### Statistical analysis

Statistical analysis of responses was descriptive only, and presented as mean, standard deviation, median and range.

## Results

Tables showing detailed summary statistics for the responses from each group (GPs, pharmacists and patients) are available in Additional file 2.

### Participant characteristics

Participant characteristics are summarised in Table 1. GPs were predominantly male and of older age, with only 10 % of respondents aged ≤50 years. Of the pharmacists, 73 % were aged ≥50 years. The mean age of patients was 32 years, with participants approximately equally split between age categories (≤20, 21–30, 31–40, over 40). The majority were office workers, students or unemployed.

**Table 1:** Participant characteristics

	General practitioners (n = 100)	Pharmacists (n = 100)	Patients (n = 552)
Age, years mean (SD)	57 (4.85)	55 (9.34)	32 (14.63)
Gender (% male)	75	54	52
Years of practice	78 % had ≥25 years’ practice	67 % had ≥25 years’ practice	N/A
Duration of AR symptoms	N/A	N/A	Mean (SD): 5.4 (8.19) years; 47 % were diagnosed with AR over 1 year ago
Mean no. of patients (SD)^a^	1287 (316) registered	3227 (2272) seen/month	N/A
Mean no. of patients with AR^a^	46 seen/month	20 % (17.95) of pts have AR	N/A

*N/A* not applicable

^a^Estimated

### Associated burden of AR

The average rating for limitation from AR on daily life given by GPs was 6.2 out of a maximum of 10, but 50 % gave a rating of 7 or 8 and 4 % a rating of 9 or 10. The average rating of limitation among patients themselves was slightly lower, at 5.7, but 30 % gave a rating of 7 or 8 and 15 % a rating of 9 or 10. When GPs were asked to rate the severity of AR in their last patient seen (on a scale of 1–10 where 1 = not at all severe and 10 = extremely severe), the average rating was 6.03 and 38 % gave a rating of 7–8, indicating that on average the last patient seen had moderate-to-severe AR.

When asked to choose which of three options was their main concern for the future with respect to AR, 61 % of patients chose ‘my health getting worse’, 28 % chose ‘limitations on my everyday life’, and 7 % chose ‘cost of treatment’ (5 % did not record a choice). This indicates that AR is a source of anxiety or concern for many patients. Fifty-nine per cent thought that AR could lead to the onset of other diseases, most notably asthma, which was mentioned by 81 % of patients who thought other diseases could develop.

### Diagnosis and symptoms

In Italy, AR appears to be diagnosed and managed mainly by GPs (in those patients who are under medical care). On average GPs estimated that they made the diagnosis personally in 68 % of cases, referred 17 % to another doctor [of whom 54 % were referred to an allergist and 39 % to an ear, nose and throat (ENT) specialist], and referred 15 % to a specialist allergy centre. Most GPs said that some patients prefer not to go a specialist centre, mainly for reasons of convenience, trust in the GP or the desire for immediate diagnosis and treatment. Of the patients surveyed, 79 % said a specialist had made the diagnosis, which was not unexpected given that participating patients were all under the care of specialist centres. Only 30 % had independently sought information on AR before diagnosis. General practitioners and internet sites were the most frequent sources of such information.

When making the diagnosis of AR, 18 % of GPs said they did not use diagnostic tests. Those who did use tests were most likely to use total serum IgE (paper radioimmunosorbent test; PRIST), specific serum IgE measurement (radioallergosorbent test: RAST) or skin prick tests; usage of other tests was negligible.

The symptoms most frequently taken into account by GPs when diagnosing AR were nasal secretion, fits of sneezing and nasal obstruction. Ocular symptoms were given substantially less significance in the diagnosis (Table 2). In contrast, tearing of the eyes was considered by pharmacists as the chief symptom that made them suspect AR, followed by the aforementioned nasal symptoms. They also cited tearing of the eyes as the most frequent symptom seen in the AR patients who consulted them. The same nasal symptoms mentioned by GPs were also most frequently mentioned by patients when asked which symptoms of AR they first experienced. Patients mentioned ocular symptoms less frequently than nasal symptoms.

**Table 2:** Symptoms considered by GPs and pharmacists when diagnosing AR, and symptoms first experienced by patients

Symptom	% citing as chief symptom		Total number of mentions		Symptoms at 1st emergence of AR (% citing)
	GPs	Pharmacists	GPs	Pharmacists	Patients
Nasal symptoms					
Nasal secretion	28	12	64	35	68
Fits of sneezing	28	24	57	41	73
Nasal obstruction	22	19	49	38	63
Itchy nose	7	9	36	29	56
Rhinorrhoea	1	5	7	8	–
Ocular symptoms					
Tearing	8	19	42	54	55
Redness of eyes	–	9	20	29	49
Conjunctivitis	–		4	–	–
Other symptoms					
Cough	1		7	3	25
Itchy palate	–	2	8	11	33
Headache	–		3		–
Respiratory problems	–	1	–	4	–
Other	2		2	4	4

Asthma was by far the most likely comorbidity to be taken into account by GPs when diagnosing AR. Of pharmacists, 8 out of 10 believed that AR is associated with underlying inflammation, and three-quarters believed that AR can cause asthma if not treated in a timely manner.

### Treatment decision-making

The GPs estimated that for 69 % of their patients they decided on the treatment themselves, while a specialist decided the treatment for 20 % and an allergy centre for 11 %. The average duration of a consultation in which a treatment was formulated for the first time was 16 min, while consultation times for patients whose treatment was already formulated were reduced to 9 min on average. In consultations involving new treatments, an average of 38 % of the time was spent framing the pathology, 23 % was spent detailing the administration method, 21 % giving reasons for proper treatment compliance, and 18 % educating the patient about any risk linked to comorbidity.

Of the patients, just over half had their current treatment prescribed by a specialist (again, to be expected given the population surveyed) and a third were using treatment prescribed by a GP. Only 4 % had their treatment recommended by a pharmacist and 4 % had chosen their own. However, pharmacists appear to play an important role in treatment decisions in the wider patient population: pharmacist respondents estimated that a third of patients they saw asked the pharmacist for a treatment recommendation. A quarter asked for a specific treatment without a prescription, and 40 % presented a doctor’s prescription.

### Treatment patterns

**Fig. 1:**
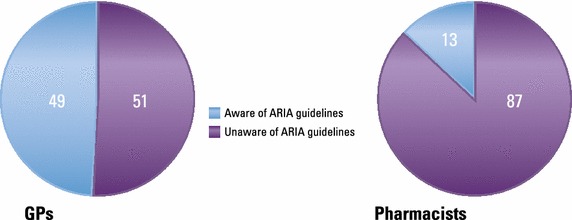
Awareness of the ARIA guidelines among GPs and pharmacists

**Fig. 2:**
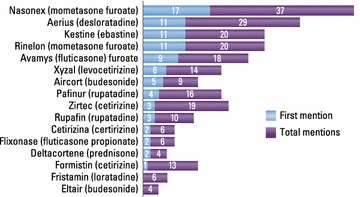
Most frequent prescribing by GPs (% mentioning product in their 3 most frequently prescribed treatments). ‘First mention’ denotes percentage who mentioned it as the product they most frequently prescribe; ‘other mentions’ denotes percentage mentioning the product as being one of the three they prescribe most frequently

Almost half of GPs (49 %) and the great majority of pharmacists (87 %) were unaware of the ARIA guidelines (Fig. 1). Of all treatments prescribed by GPs, 57 % were oral medications and 46 % nasal medications. About half were ongoing treatments and half were for use as needed. When asked which three products they prescribed most frequently for AR, GPs mentioned branded mometasone furoate most frequently, followed by desloratadine, ebastine and generic mometasone (Fig. 2). Antihistamines were mentioned more frequently than intranasal steroids (INSs). Twenty-one percent of GPs said they sometimes prescribed homeopathic products.

**Fig. 3:**
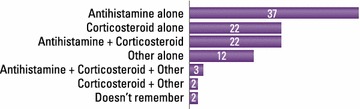
Prescribing by GPs for last AR patient seen (% prescribing product)

For the ‘last patient’ case, which had moderate-to-severe AR on average, 37 % of GPs reported prescribing antihistamine alone and 27 % prescribed two products, most commonly an antihistamine and an INS (Fig. 3). For ‘last patient’ cases who were already on treatment, GPs reported that in 78 % of cases they did not change the prescription, which was an antihistamine in the majority of cases.

The pharmacist and patient surveys provided additional perspectives on treatment patterns. Pharmacists reported recommending an antihistamine for most cases (56 %) who asked for a recommendation, followed by a vasoconstrictor (21 %), homeopathic therapy (12 %) or an intranasal/oral corticosteroid (12 %). They estimated that 55 % of doctors’ prescriptions they dealt with were for antihistamines, 37 % for intranasal or oral corticosteroids, and 15 % for vasoconstrictors. Half of independent requests by patients were estimated to be for antihistamines, and a quarter each for INS and vasoconstrictors. These trends were broadly reflected in the ‘last patient’ case responses (data not shown).

**Fig. 4:**
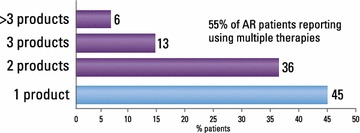
Proportion of patients using multiple therapies

When patients were asked what products they currently used to control their AR, 69 % said they used an antihistamine and 25 % an INS, which was in agreement with the GP and pharmacist surveys. However, only 4 % used vasoconstrictors and 3 % homeopathy. Nine per cent used other remedies and 17 % were not using any treatment. A quarter said they would consider using homeopathy. On average, patients were using 1.7 products, and 55 % reported using multiple therapies (Fig. 4). The most common combination was an antihistamine and an INS.

### Satisfaction with treatment

**Fig. 5:**
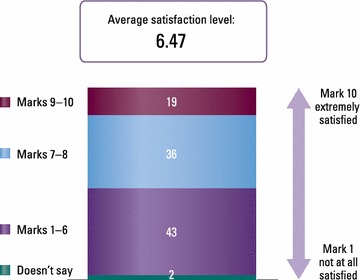
Patient satisfaction with current AR treatment

Just over 40 % of patients were either not satisfied or weakly satisfied with their current treatment (satisfaction rating of ≤6 out of 10), with an average rating of 6.47 (Fig. 5). Satisfaction with the way healthcare professionals had managed their AR to date was somewhat higher (average rating 7.25). Patients’ main expectation for the future was to succeed in managing or easing their AR symptoms (45 %), while 22 % hoped for a definitive cure.

## Discussion

This survey details for the first time the practices of GPs and pharmacists in relation to diagnosis and treatment of AR in Italy. It also gives significant insight into patients’ behaviours and attitudes to this prevalent condition and its treatment.

Allergic rhinitis is on the increase [21] and becoming ever more challenging to treat so it is more important than ever that HCPs have the most up-to-date evidence on the best treatments. The ARIA guidelines [7] set out evidence-based standards of best practice for diagnosis and treatment of AR. However, the current survey suggested that only half of GPs in Italy are aware of these guidelines, despite seeing an average of 46 AR patients a month. Awareness is even lower among community pharmacists, at just 13 %, even though participating pharmacists estimated that 20 % of their clients had AR. There are specific ARIA guidelines for pharmacists detailing all aspects of AR diagnosis and treatment, with advice given on when referral to a physician is appropriate [22]. Clearly, more needs to be done to make HCPs aware of the guidelines for diagnosing and treating this extremely common condition.

Allergic Rhinitis and its Impact on Asthma recommends that patients who present with two or more symptoms of AR (rhinorrhoea, sneezing, nasal obstruction/pruritus ± conjunctivitis) for >1 h a day, and who have a positive allergen test, should be referred to a specialist for diagnosis and disease classification (e.g. intermittent, persistent, non-allergic) [6, 23]. However, the survey showed that in Italy it is GPs, not allergy specialists, who are making the majority of AR diagnoses and formulating the treatment plan, either when the patient presents directly or is referred by a pharmacist. Only a third of patients presenting to GPs with possible AR were referred to specialist doctor or allergy centres. One reason for this referral deficit is that some patients prefer the convenience and immediacy of diagnosis and treatment by their GP and do not wish to visit a specialist.

With regard to treatment, the ARIA algorithm recommends a step-up, step-down approach depending on whether the symptoms are intermittent or persistent. Antihistamines and leukotriene-receptor antagonists (LTRA) are recommended as first-line for patients with mild symptoms, while INSs are considered first-line for patients with moderate-to-severe AR [7, 23]. This survey revealed a significant mismatch between treatment patterns among Italian GPs and pharmacists and the ARIA recommendations. GPs perceived that the average patient presenting to them had moderate-to-severe AR, yet the most frequently prescribed AR treatment was an antihistamine alone, which is not concordant with the ARIA guidelines. Judging by the fact that 69 % of patients reported using an antihistamine and 25 % an INS, and just over half said their treatment had been prescribed by a specialist, there also appears to be some deviation from the ARIA guidelines among specialists, although the prescribing patterns of specialists were not surveyed directly, and neither was the AR severity of the patients. There was also a reluctance by GPs to change AR treatment even if the patient had moderate-to-severe symptoms remaining. This may reflect the fact that oral antihistamines are reimbursed for chronic AR throughout Italy, whereas only one region (Tuscany) currently reimburses nasal corticosteroids. Nasal antihistamines are not reimbursed so are not widely used in Italy. Pharmacists too recommended antihistamines most frequently, although this may reflect a milder degree of symptoms in those patients seeking treatment without a doctor’s prescription. There was also some confusion among pharmacists about the principal symptoms of AR. Eye watering was cited above nasal symptoms as the main symptom leading to suspicion of AR, even though it is not classified as a major indicator of AR in the guidelines. Pharmacists reported that they referred just under half of patients to a doctor.

Despite reporting satisfaction with the management of their AR by doctors and pharmacists, patients in Italy are less happy with AR treatments. The average satisfaction rating was reported as 6.4 out of 10, with 43 % of patients reporting a rating of 6 or below. One reason for the low satisfaction may be the knowledge base from which GPs and pharmacists are working, and therefore prescribing. However, AR is known to be a difficult condition to treat. It is likely that patients’ dissatisfaction also stems from the therapeutic deficits of commonly prescribed therapies; participating patients were using an average of 1.7 therapies and 55 % of patients were on more than one treatment. Adding an oral antihistamine or LTRA to an INS is neither officially recommended by the guidelines [7] nor is it supported by the scientific evidence [24, 25].

Patients have a significant impact on their own AR management. Many AR medications are over the counter and patients frequently self-diagnose. Pharmacists reported that 1 in 4 patients asked for a specific treatment, and that it was most often an antihistamine. Patients also decide whether or not to adhere to prescribed medication. Perhaps surprisingly, GPs and pharmacists on average perceived a slightly greater degree of limitation to daily life from AR symptoms than was reported by patients themselves, even though the patients recruited were under the care of specialist centres. This suggests that failure by HCPs to take AR seriously is not a major issue in Italy. However, 15 % of patients gave a limitation rating of 9 or 10, showing that for a minority of sufferers AR imposes a very severe limitation on everyday activities.

What can be done to improve the management of AR in Italy and elsewhere? It is clear that a greater awareness of formal guidelines for treatment would be beneficial for all parties. It would enable GPs to assess disease severity with greater accuracy and to prescribe the most effective treatment options for individual patients. For pharmacists, it is important to be able to accurately recognise AR and assess its severity, and make a guideline-informed treatment recommendation, whether it is medication or referral to a physician.

To close the gap between current practices and the most up-to-date evidence-based treatment strategies, allergy societies and guidelines need to adapt to the reality that AR is diagnosed and managed by generalist physicians in the majority of cases. Assessment of AR needs to be standardised and simplified, for example by using a visual analogue scale (VAS). This would likely lead to improved prescribing for moderate-to-severe disease. Patients on multiple therapies and those whose symptoms are insufficiently controlled on INS or intranasal antihistamine monotherapy could benefit from increased awareness of new and more effective AR treatments, which could potentially increase treatment satisfaction through improved convenience and more efficient drug delivery [26]. For example, an advanced intranasal delivery system of azelastine hydrochloride and fluticasone propionate (Dymista^®^, Meda, Solna, Sweden) is now available in Italy which is twice as effective as an INS and provides rapid symptom control in real-life [27–31]. Improved communication between HCPs and patients regarding degree of symptom control is also needed, so that GPs can more easily recognise dissatisfaction with treatment and are encouraged to change a patients’ treatment if it is not providing adequate control. Progress is being made in this direction: for example, ARIA in collaboration with MACVIA-LR (a reference site of the European Innovation Partnership on Active and Health Aging) is currently developing a digital VAS-based app for patients to document AR symptom control on therapy [32]. Companion apps for pharmacists and GPs are also planned with the aim of introducing a common language of AR control for all stakeholders and ultimately improving inter-discipline and patient communication and the way AR is assessed and treated.

## Conclusion

Allergic rhinitis is largely managed by GPs in Italy. Pharmacists are also important, acting as gatekeepers to treatment. Awareness of the ARIA guidelines among GPs and pharmacists is low, and treatment is not always in accordance with evidence-based best practice. Patient satisfaction with treatment is moderate or low. Allergy education programs need to be targeted at GPs and pharmacists, and communication with patients regarding symptom control needs to be improved.

## Additional files

**Additional file 1:** Survey wording (general practitioner, community pharmacist and patient surveys).
**Additional file 2:** Summary statistics of the responses for each group (GPs, pharmacists and patients).

## Abbreviations

AR: allergic rhinitis
ARIA: Allergic Rhinitis and its Impact on Asthma
ENT: ear, nose and throat
GP: general practitioner
HCP: healthcare provider
INS: intranasal steroid
OTC: over the counter
PRIST: paper radioimmunosorbent test
RAST: radioallergosorbent test
VAS: visual analogue scale
