# Supplementary material for: 360 degree perspective on allergic rhinitis management in Italy: a survey of GPs, pharmacists and patients
Source: Clin Mol Allergy. 2015 Nov 2;13:25. doi: 10.1186/s12948-015-0029-5 (PMC4629286; doi:10.1186/s12948-015-0029-5)
Supplement: Supplementary file 1 — Additional file 1: Survey wording (general practitioner, community pharmacist and patient surveys). [file 12948_2015_29_MOESM1_ESM.pdf]

Survey wording (general practitioner, community pharmacist and patient surveys)

GP survey:

1. Are you a currently practicing GP?
    1. Yes
    2. No
2. Doctor, how many total patients do you have?
3. And of these patients, in an average month how many suffer from Allergic Rhinitis?
4. Out of a total of 100 of your patients with Allergic Rhinitis for what percentage did you make the diagnosis personally, what percentage did you refer to another physician, and what percentage did you send to a specialized centre for Allergic Rhinitis?
    1. I made the diagnosis personally (%)
    2. I referred the patient to another physician (%)
    3. I sent the patient to an allergy centre

a. To what physician in particular do you send your patients?

- c. To what Allergology centre do you send the patients?
- e. Are there any patients that prefer to not turn to the Specialized Centres and instead want a remedy from you?
    1. Yes
    2. No
- f. For what reason in your opinion?

5. What is the chief symptom you take into consideration to diagnose Allergic Rhinitis (single answer)?
    1. Cough
    2. Nasal secretion
    3. Nasal obstruction

    4. Fits of sneezing
    5. Nasal itch
    6. Palatal itch
    7. Watery eyes
    8. Red eyes
    9. Other (specify)

6. And what other symptoms do you take into account (multiple answers possible)?
    1. Cough
    2. Nasal secretion
    3. Nasal obstruction
    4. Fits of sneezing
    5. Nasal itch
    6. Palatal itch
    7. Watery eyes
    8. Red eyes
    9. Other (specify)
7. Doctor, in the cases for whom you make the diagnosis of Allergic Rhinitis personally, what diagnostic tests do you prescribe?
    1. Rhinoscopy
    2. Skin prick test
    3. PRIST, total serum IgE
    4. RAST, antigen specific IgE
    5. Nasal cytology study (Studio del secreto nasale, citologia e ricerca di IgE)
    6. Other (specify)
8. What are the main pathologies correlated to Allergic Rhinitis that you take into consideration in the phase of diagnosing your patients?

9. And in your opinion, what comorbidities are the patients affected by Allergic Rhinitis subject to?
    1. Asthma
    2. Conjunctivitis
    3. Polyposis
    4. Sinusitis
    5. Otitis media
    6. Other (specify)
10. Out of 100 patients you see with Allergic Rhinitis, for how many did you formulate the treatment personally, for how many was it formulated by a specialist, and for how many by a Specialized Centre?
    1. I formulated the treatment personally
    2. It was formulated by a specialist
    3. It was formulated at a specialist centre
11. Out of 100 patients with Allergic Rhinitis you treat, what percentage of these are new patients (first treatment) and how many are already in treatment?
    1. New patients (%)
    2. Already in treatment (%)
12. Doctor, on average how much time do you devote to visiting a patient for whom you are setting up treatment for the first time?
    1. Minutes
    2. Hours
        a. More specifically, of the time you told me, what percentage do you devote to:
    1. Framing the pathology
    2. Treatment administration method
    3. Reasons for proper compliance with treatment
    4. Any risks linked to comorbidity

13. On average, how much time do you devote to the visit of a patient with a treatment already formulated?
    1. Minutes
    2. Hours
14. Now think about the total of your patients in treatment for Allergic Rhinitis. What 3 products do you prescribe most frequently?
15. Out of 100 treatments you prescribe, to what percentage of cases do you prescribe products administered orally and to what percentage products administered by nose?
    1. Oral (%)
    2. Intranasal (%)
        a. And again out of 100 treatments prescribed by you, what percentage are ongoing treatments and what percentage as needed?
        1. Ongoing (%)
        2. As needed (%)
16. Do you prescribe/recommend homeopathic products for the treatment of Allergic Rhinitis? If yes see 17
    1. Yes
    2. No
17. Out of 100 of your patients in treatment for Allergic Rhinitis, to what percentage do you prescribe/recommend a homeopathic product?
18. Are you aware of the A.R.I.A. guidelines?
    1. Yes
    2. No
19. On a scale from 1 to 10 (where 1 = not at all and 10=very) IN YOUR PROFESSIONAL EXPERIENCE, how much does Allergic Rhinitis limit the everyday life of the patients?

GP last patient case:

1. What is the patient's age?
2. What is the patient's gender?
    1. Man
    2. Woman
3. Did you personally make the first diagnosis of Allergic Rhinitis in this patient?
    1. Yes (see 4)
    2. No (see 5)
4. Who sent the patient to you?
    1. Pharmacist
    2. Medical specialist (specify)
    3. Specialist AR centre
5. What symptoms did the patient present?
    1. Cough
    2. Nasal secretion
    3. Nasal obstruction
    4. Fits of sneezing
    5. Nasal itch
    6. Palatal itch
    7. Watery eyes
    8. Red eyes
    9. Other (specify)
6. And what tests did you prescribe?
    1. Rhinoscopy
    2. Skin prick test
    3. PRIST, total serum IgE
    4. RAST, antigen specific IgE

   5. Nasal cytology study (Studio del secreto nasale, citologia e ricerca di IgE)
   6. Other (specify)
7. On a scale from 1 to 10, where 1 = not severe at all and 10=very severe, what was the level of severity of this patient's Allergic Rhinitis?
8. Did the patient present concomitant diseases? If so, what ones?
9. What treatment did you prescribe to this patient?
   a. And does the patient take other products as well?
      1. Yes
      2. No
   b. And what product in particular (please include the brand)?
10. What was the duration of the therapy?
    1. Days
    2. Weeks
    3. Years
11. Was the patient treating the Allergic Rhinitis for the first time or was he/she already in treatment?
    1. Patient in treatment for the first time
    2. Patient already receiving treatment
12. And was the patient in treatment with the same drug, or did he change treatment?
    1. Patient in treatment with the same drug
    2. Patient changed treatment
13. What was the previous treatment (please include the brand)?
14. Is a follow-up visit anticipated for this patient?
    1. Yes (see 15)
    2. No
15. When will this visit be?

1. Days
2. Weeks
3. Years

Other data

A. Sex
    1. Man
    2. Woman
B. Age
C. Years a professional (Anzianità professionale)
    1. Less than 5 years
    2. 5 to 10 years
    3. 11 to 15 years
    4. 16 to 20 years
    5. 21 to 25 years
    6. More than 25 years
D. Region
    1. Piemonte
    2. Valle d'Aosta
    3. Lombardia
    4. Liguria
    5. Friuli V.G.
    6. Trentino A.A.
    7. Veneto
    8. Emilia Romagna
    9. Toscana
    10. Marche
    11. Lazio
    12. Umbria
    13. Abruzzo
    14. Molise
    15. Campania
    16. Puglia
    17. Basilicata
    18. Calabria
    19. Sicilia
    20. Sardegna
E. Centre of residence

1. Capital of province
2. Outside capital of province

F. Population

1. Less than 30,000
2. 30,000 to 100,000
3. 100,000 to 500,000
4. Greater than 500,000

Pharmacist survey

1. Do you currently practice as a pharmacist? If yes, see 2
    1. Yes (see a)
    2. No
    a. What role do you play in the pharmacy?
        1. Proprietor
        2. Manager
        3. Collaborator
        4. Other (please specify)
2. In the course of an average month, how many patients enter your pharmacy?
3. Out of 100 of these patients, considering the seasonal nature of AR and the possibility of having months of high morbidity and low morbidity, what percentage in your opinion suffer from Allergic Rhinitis?
4. What is the main symptom that makes you suspect the presence of Allergic Rhinitis in your pharmacy's clients (single answer)?
    1. Cough
    2. Nasal secretion
    3. Nasal obstruction
    4. Fits of sneezing
    5. Nasal itch
    6. Palatal itch
    7. Watery eyes
    8. Red eyes
    9. Other (specify)
5. And what other symptoms (multiple answers possible)?
    1. Cough
    2. Nasal secretion
    3. Nasal obstruction

4. Fits of sneezing
5. Nasal itch
6. Palatal itch
7. Watery eyes
8. Red eyes
9. Other (specify)

6. In your view, does Allergic Rhinitis have an underlying inflammation?
   1. Yes
   2. No
7. And in your opinion, can Allergic Rhinitis cause asthma if not treated in a timely manner?
   1. Yes
   2. No
8. Considering the seasonal nature of RA and the possibility of having months of high morbidity and low morbidity, relative to an average month that takes these variations into account, how many patients with Allergic Rhinitis turn to you?
9. And what symptoms do these patients present?
   1. Cough
   2. Nasal secretion
   3. Nasal obstruction
   4. Fits of sneezing
   5. Nasal itch
   6. Palatal itch
   7. Watery eyes
   8. Red eyes
   9. Other (specify)

10. Out of 100 patients with Allergic Rhinitis who turn to you, what percentage of them ask you for a recommendation, how many a specific treatment and how many present a doctor's prescription?
    1. Ask for a recommendation (%)
    2. Ask for a specific treatment (%)
    3. Have a doctor's prescription (%)
11. To those who asked for advice, to how many of them did you suggest…
    1. Antihistamine (%)
    2. Intranasal or oral corticosteroid (%)
    3. Homeopathic product (%)
    4. Vasoconstrictor (%)
    5. Other treatment (specify) (%)

    a. In how many cases are the products…

    1. Oral (%)
    2. Intranasal (%)
12. And again, out of 100 of the patients with Allergic Rhinitis who ask you for a recommendation, to what percentage of them do you suggest going to see the doctor?
13. And generally to what physician do you refer the patient?
    1. GP
    2. Medical specialist (specify)
    3. Allergology centre
14. Out of 100 of the patients with Allergic Rhinitis who asked you for a specific treatment, what percentage asked you for...?
    1. Antihistamine (%)
    2. Oral or intranasal corticosteroid (%)
    3. Homeopathic product (%)
    4. Vasoconstrictor (%)

5. Other (specify)

a. Out of 100 of the specific treatments requested by your patients for the treatment of Allergic Rhinitis, in what percentage of cases are they products taken orally and in what percentage are they products taken by nose?

1. Oral (%)
2. Intranasal (%)

15. Out of 100 of the patients with Allergic Rhinitis who presented a doctor's prescription to you, in what percentage of cases was it for...?

    1. Antihistamine (%)
    2. Oral or intranasal corticosteroid (%)
    3. Homeopathic product (%)
    4. Vasoconstrictor (%)
    5. Other (specify)

    a. Out of 100 of the prescriptions presented by your patients for the treatment of Allergic Rhinitis, in what percentage of cases were they products for oral administration and in what percentage products to administer directly to the nose?

    1. Oral (%)
    2. Intranasal (%)

16. And in particular, what product is the most prescribed for the treatment of Allergic Rhinitis?
17. And what others?
18. As far as you know, are there any Allergy Centres in your city?

    1. Yes
    2. No

19. Are there any patients who prefer to NOT turn to Specialized Centres and instead want to get a remedy from you?

1. Yes
2. No

20. For what reasons in your opinion are there patients who prefer to NOT turn to the Specialized centres and instead want to get a remedy from you?
21. Are you aware of the A.R.I.A. guidelines?
    1. Yes
    2. No

Other data

A. Sex
    1. Man
    2. Woman
B. Age
C. Years a professional (Anzianità professionale)
    1. Less than 5 years
    2. 5 to 10 years
    3. 11 to 15 years
    4. 16 to 20 years
    5. 21 to 25 years
    6. More than 25 years
D. Region
    1. Piemonte
    2. Valle d'Aosta
    3. Lombardia
    4. Liguria
    5. Friuli V.G.
    6. Trentino A.A.
    7. Veneto
    8. Emilia Romagna
    9. Toscana
    10. Marche
    11. Lazio
    12. Umbria
    13. Abruzzo
    14. Molise

15. Campania
16. Puglia
17. Basilicata
18. Calabria
19. Sicilia
20. Sardegna

E. Centre of residence
    1. Capital of province
    2. Outside capital of province

F. Population

1. Less than 30,000
2. 30,000 to 100,000
3. 100,000 to 500,000
4. Greater than 500,000

G. Type of pharmacy

1. Urban pharmacy
2. Rural pharmacyW

Pharmacist last patient case:

1. What is the patient’s age?
2. What is the patient’s gender?
    1. Man
    2. Woman
5. What symptoms did the patient present?
    1. Cough
    2. Nasal secretion
    3. Nasal obstruction
    4. Fits of sneezing
    5. Nasal itch
    6. Palatal itch
    7. Watery eyes
    8. Red eyes

    9. Other (specify)

7. On a scale from 1 to 10, where 1 = not severe at all and 10 = very severe, what was the level of severity of this patient's Allergic Rhinitis?
8. Did this patient ask you for a recommendation, a treatment, or did he come in with a doctor's prescription?
    1. Ask for a recommendation
    2. Ask for a specific treatment
    3. Have a doctor's prescription
9. What did you recommend to the patient?
    1. Antihistamine
    2. Intranasal corticosteroid
    3. Oral corticosteroid
    4. Homeopathic product
    5. Vasoconstrictor
    6. To see a doctor
    7. Other (specify)
10. What specific treatment did the patient ask you for?
    1. Antihistamine
    2. Corticosteroid
    3. Homeopathic product
    4. Vasoconstrictor
    5. Other (specify)
11. What type of product was the prescription for?
    1. Antihistamine
    2. Intranasal corticosteroid
    3. Oral corticosteroid
    4. Homeopathic product
    5. Vasoconstrictor

6. Other (specify)

Patient survey

1. When were you first diagnosed with allergic rhinitis?
    1. Today
    2. Days ago
    3. Months ago
    4. Years ago
2. With what symptoms did your Allergic Rhinitis emerge?
    1. Cough
    2. Nasal secretion
    3. Nasal obstruction
    4. Fits of sneezing
    5. Nasal itch
    6. Palatal itch
    7. Watery eyes
    8. Red eyes
    9. Other (specify)
3. How long were you suffering with allergic rhinitis before a diagnosis was made?
    1. Days
    2. Months
    3. Years
4. Who first suspected your Allergic Rhinitis?
    1. Pharmacist
    2. GP (Medico di base?)
    3. Specialist
    4. Yourself
    5. Other (specify)
5. And who made the definite diagnosis of Allergic Rhinitis?

    1. Pharmacist
    2. GP (Medico di base?)
    3. Specialist
    4. Yourself
    5. Other (specify)
6. Before receiving the diagnosis, had you gathered information on Allergic Rhinitis on your own?
    1. Yes
    2. No (see 8)
7. And where in particular did you seek information on the Allergic Rhinitis from which you suffer?
1. Health information websites
2. Allergic rhinitis websites
3. Forums and social networks
4. Scientific publications
5. Newspaper/TV/radio
6. GP (Medico di base?)
7. Pharmacist
8. Family/friends
9. Other (specify)
10. Did you take the possibility of treating yourself with homeopathic remedies into consideration?
    1. Yes
    2. No (see 10)
11. Who prescribed\recommended a homeopathic remedy to you?
    1. GP (Medico di base)
    2. Pharmacist
    3. Specialist (specify)

4. Family/friends
5. I chose it independently
6. Other (specify)

12. With what type of product do you currently treat your allergic rhinitis?
    1. Antihistamine
    2. Corticosteroid (un cortisone)
    3. Homeopathic product
    4. A vasoconstrictor
    5. Other remedy (specify)
    6. Nothing (See 16)
13. And do you take the treatment regularly or as needed?
    1. Regularly
    2. As needed
14. And is it a product taken by mouth or nose?
    1. Oral
    2. Intranasal
15. Who prescribed\recommended the treatment for Allergic Rhinitis you are following at present?
    1. GP (Medico di base)
    2. Pharmacist
    3. Specialist (specify)
    4. Family/friends
    5. I chose it independently
    6. Other (specify)
16. How many drugs/products do you take for your allergic rhinitis?
17. On a scale from 1 to 10, as of today how satisfied are you with the therapy you are following to treat the Allergic Rhinitis you suffer from?

18. Now think about all the professional figures you saw during the process of diagnosing and treating the Allergic Rhinitis from which you suffer. On a scale from 1 to 10 (where 1= not satisfied at all and 10= very satisfied) how satisfied are you, to date, with how the Allergic Rhinitis from which you suffer has been managed?
19. Your degree of satisfaction aside, in your view was anything missing in how your Allergic Rhinitis was handled?
20. Who advised\suggested that you turn to a specialized centre for the treatment of Allergic Rhinitis?
    1. GP (Medico di base)
    2. Pharmacist
    3. Specialist (specify)
    4. Family/friends
    5. I chose it independently
    6. Other (specify)
21. On a scale from 1 to 10, in your view how much does the Allergic Rhinitis from which you suffer limit your everyday life?
22. What is your main expectation for the future of your allergic rhinitis treatment?
23. And what instead is your main concern for the future with respect to your Allergic Rhinitis?
    1. The deterioration of my health
    2. The limiting effects on my daily life
    3. The cost of treatment
24. In your view, could the Allergic Rhinitis from which you suffer lead to the development or onset of other pathologies?
    1. Yes
    2. No
25. And what pathologies in particular?

1. Asthma
2. Conjunctivitis
3. Polyposis
4. Sinusitis
5. Otitis
6. Other (specify)

Other data

A. Sex
    1. Man
    2. Woman
B. Age
C. What qualification do you hold?
    1. Elementary school (lizenca elementare)
    2. Lower diploma (Diploma di media inferior)
    3. Vocational school diploma
    4. Higher diploma (Diploma di media superiore)
    5. Degree
    6. Master/postgraduate/doctorate
D. What is your current employment status
    1. Employee
    2. Dealer/operator
    3. Trade unionist/ worker representative
    4. Lecturer/teacher
    5. Unemployed
    6. Housewife
    7. Retired
    8. Freelance
    9. Businessman/woman

10. Manager

E. Region

1. Piemonte
2. Valle d’Aosta
3. Lombardia
4. Liguria
5. Friuli V.G.
6. Trentino A.A.
7. Veneto
8. Emilia Romagna
9. Toscana
10. Marche
11. Lazio
12. Umbria
13. Abruzzo
14. Molise
15. Campania
16. Puglia
17. Basilicata
18. Calabria
19. Sicilia
20. Sardegna

F. Centre of residence

    3. Capital of province

    4. Outside capital of province

G. Population

    1. Less than 30,000

    2. 30,000 to 100,000

    3. 100,000 to 500,000

    4. Greater than 500,000
